# Sp1-Induced lncRNA Rmrp Promotes Mesangial Cell Proliferation and Fibrosis in Diabetic Nephropathy by Modulating the miR-1a-3p/JunD Pathway

**DOI:** 10.3389/fendo.2021.690784

**Published:** 2021-08-27

**Authors:** Hansen Yang, Jia Wang, Zheng Zhang, Rui Peng, Dan Lv, Handeng Liu, Yan Sun

**Affiliations:** ^1^Department of Cell Biology and Genetics, Chongqing Medical University, Chongqing, China; ^2^Department of Bioinformatics, Chongqing Medical University, Chongqing, China

**Keywords:** diabetic nephropathy, Rmrp, mesangial cells, miR-1a-3p, JunD

## Abstract

Diabetic nephropathy (DN) is a serious complication of diabetes mellitus. Long non-coding RNAs (lncRNAs) are regulators in DN progression. However, the regulatory mechanisms of multiple lncRNAs in DN remain to be determined. Our aim was to investigate the function and molecular mechanism of lncRNA RNA component of mitochondrial RNAase P (Rmrp) in DN. Here, we observed that the expression of Rmrp was up-regulated in the kidney of db/db DN mice and high glucose induced glomerular mesangial cells (MC). More importantly, the abnormal transcription of Rmrp was induced by nuclear transcription factor Sp1, which promotes the proliferation and production of fibrotic markers in MC. Subsequently, we screened the miRNAs related to Rmrp and found that Rmrp and miR-1a-3p are co-localized at the subcellular level of MC, and Rmrp could directly binds to miR-1a-3p. Further mechanism research demonstrated that the elevated miR-1a-3p significantly attenuated the proliferation and fibrosis-promoting effects induced by up-regulation of Rmrp. At the same time, we also investigated that miR-1a-3p can directly bind to Jun D proto-oncogene (JunD), thereby regulating the protein level of JunD. Rmrp-induced proliferation and fibrogenesis were reversed by co-transfection with JunD siRNA. In summary, Sp1 induced lncRNA Rmrp could drive the expression of JunD *via* sponging miR-1a-3p in DN progression.

## Introduction

The increasing global prevalence of diabetic nephropathy (DN) is closely associated with end-stage renal disease (1, 2). Glomerular mesangial enlargement is the basic pathophysiological mechanisms of DN and a recognized feature in the early of DN process. This lesion in mesangial tissue is initially due to the mesangial cells (MC) proliferation and extracellular matrix (ECM) accumulation (3, 4). Although hyperglycemia is the main determinant in the mesangial change of DN, the current interventions and medication are not ideal (5). Therefore, it is an urgent need to illustrate the mechanisms of DN.

Long non-coding RNAs (lncRNAs) are identified as RNAs>200 nucleotides and play important regulatory roles in many diseases (6, 7). In the past decade, researchers demonstrate lncRNAs that play key modulators in DN pathogenesis. For instance, LRNA9884 could promote renal inflammation by regulating MCP-1 transcriptional level in DN mice (8). In addition, a recent study also found that glomerular ECM and hypertrophy are regulated by lncRNA lncMGC, which is provoked by endoplasmic reticulum stress (9). Nevertheless, the potential roles, biogenesis process and molecular functions of lncRNAs in DN are still largely unclear.

MicroRNAs (miRNAs) as small non-coding RNAs are demonstrated to play an important agent in cellular processes and molecular mechanism of the disease state, including DN (10, 11). Many miRNAs have been exhibited to a negative effect on gene expression and function at the transcriptional and post-transcriptional regulation. One classic hypothesis of miRNA function is the competitive endogenous RNA (ceRNA) hypothesis that RNA transcripts can band to miRNA-complementary sequence and regulate each other by competing specifically for shared miRNAs (12). Mounting evidence shows that lncRNAs could act as ceRNAs to bind with miRNA and free its target genes for translation in DN (13). It has been reported that lncRNA-NR_033515 could promote MC proliferation and trigger fibrogenesis-related proteins by targeting miR-743b-5p in DN (14). Our previous study also showed lncRNA H2k2 could serve as ceRNA activates Trim11/Mek pathway *via* binding the miR-449a/b and promotes mesangial cell proliferation in DN (15).

By using high-throughput RNA-seq, we recently reported the involvement of lncRNAs in the kidney of DN mice and identified lncRNAs associated with DN (16, 17). In the current study, we found that lncRNA RNA component of mitochondrial RNAase P (Rmrp) is the high expressed lncRNAs in the DN mice and high glucose induced-MC. Rmrp as a non-coding RNA (ncRNA), is located on mouse chromosome 4 (Chr4: 43492785-43493059) and having a length of 275 bp, and is a part of the RNase MRP complex functioning in mitochondrial and ribosomal RNA processing (18). The full-length Rmrp is highly conserved between human and mouse (84% homology) (19). However, there is no report about Rmrp in DN up to now. More importantly, we found that transcription factor Sp-1 might mediate the transcription of Rmrp. We further proved that Rmrp up-regulated JunD expression through sponge miR-1a-3p, which may contribute to MC proliferation and fibrosis in DN. Collectively, Our results provide novel insight into the functions of RMRP in DN.

## Materials and Methods

### Animal Tissue Specimens

The kidney tissue stripped from C57BL/BKS background db/db male mice (Lepr^db^/+Lepr^db^) or age-matched male genetic control db/m mice (m+/+ Lepr^db^), that were purchased from Nanjing Biomedical Research Institute (Nanjing, China). As we previously published (16), the levels of blood glucose, albuminuria and creatinine were detected in db/db mice and db/m mice, and the 8 weeks of age of db/db mice were regarded as the early stage of DN. The tissue specimens were stored in liquid nitrogen until further use. All procedures follow Chongqing Medical University’s facility guidelines for animal experiments and management. The study was approved by the Ethics Committee of Chongqing Medical University.

### Cell Culture

Mouse glomerular endothelial cell (MGEC), glomerular mesangial cells (MC), glomerular podocyte cells (MPC5) and renal tubular epithelial cells (TCMK-1) were preserved in our laboratory. MPC5 were cultured in RPMI 1640 medium (Invitrogen, CA, USA). MGEC, MC and TCMK-1were cultured in DMEM medium (Gibco, CA, USA). As previously reported (17), these cells were stimulated with glucose at 5.5 mmol/L glucose and 19.5 mmol/L mannitol (low glucose MC, LMC) or at 25 mmol/L glucose (high glucose MC, HMC).

### Vector Construction, Small Interfering RNA, and Cell Transfection

The full length of Rmrp was amplified in MCs and was cloned into the lentivirus vector LV5 (GenePharma, Shanghai, China). The full length of Sp1 was amplified from cDNA and was cloned into pcDNA3.1 (Genecreate Biotech, Wuhan, China). Vectors were identified by enzyme digestion identification (Rmrp over-expression lentivirus digested by BamHI and NotI; Sp-1 over-expression plasmid digested by BamHI and EcoRV). The empty sequence (NC) of LV5 and pcDNA3.1 vector was used as controls, respectively. Small interfering RNAs (siRNAs) targeting Rmrp were designed and purchased by Ribo Biotech (Guangzhou, China); JunD siRNAs, Ap-1siRNAs and negative control (siNC) were designed and purchased by Sangon Biotech (Shanghai, China). MiR-1a-3p mimic, inhibitor and matched negative controls were provided by GenePharma. Lentivirus containing Rmrp were generated in 293T cells. MCs were infected with the lentivirus and selected with puromycin (Gibco). The transfections were performed with Lipofectamine 3000 (Invitrogen) according to the manufacturer’s instructions. The sequences of siRNA, mimics, inhibitor and negative control in this study were listed in **Supplementary Table 1**.

### RNA Extraction, Nuclear-Cytoplasmic Fractionation, and qRT-PCR Assays

All the processes were executed with the manufacturer’s instructions. Cytoplasmic and nuclear RNA were isolated by PARIS™ Kit (Invitrogen). TRIzol^®^ Reagent (Invitrogen) was used to extract total RNA from tissues or cell. The PrimeScript RT kit (Takara, Dalian, China) was applied to the reverse transcription of RNA. The TB Green Premix Ex Taq (Takara) and CFX Connect™ Real-Time PCR Detection System (Bio-Rad, CA, USA) were used to perform the quantitative real-time PCR (qRT-PCR). Relative expression changes of genes were calculated by the 2^−ΔΔCt^ method. All the primer sequences were displayed in **Supplementary Table 2**.

### Fluorescence *In Situ* Hybridization

Cy3-labeled Rmrp and FITC-labeled miR-1a-3p fluorescence probe were synthesized by GenePharma (Shanghai, China). FISH analysis was performed using a FISH kit (GenePharma, Shanghai, China) according to the manufacturer’s instructions. The images were observed with the fluorescence microscope (Leica, Wetzlar, Germany) and analyzed with Image-Pro Plus (Media Cybernetics, Bethesda, MD, USA).

### Immunohistochemistry

The detailed method was performed as previously reported (20). Briefly, Paraffin-embedded renal tissues were sectioned at 4 µm thickness. Antigen retrieval was used with citrate buffer for 15 min in a microwave oven at 95–98°C. Endogenous peroxidase of renal tissues was occluded by 3% H_2_O_2_ for 20 min at room temperature. Next, the sections were blocked with 0.5% Triton and 10% normal goat for 30 min at room temperature and then incubated with anti-JunD (1:100, Abcam) at 4°C for 12 h. Subsequently, the sections were washed with PBS three times and carried out with biotinylated secondary antibody at room temperature for 30 min. The diaminobenzidine (Zhongshan Biosciences, Beijing, China) was applied to the chromogenic reaction and hematoxylin was used to stain the nuclei for the sections. The immunohistological images were observed by light microscopy (Leica).

### Cell Proliferation and Flow Cytometric Analysis

Cell proliferation rate was measured by 5-ethynyl-2’-deoxyuridine (EdU) assays according to the manufacturer’s instructions. Flow cytometric analysis was performed as previously reported (15).

### Western Blot Analysis

The proteins were extracted by cold RIPA lysis buffer, quantified and electrophoresed by SDS-PAGE (8, 10, or 12%). Next, the target protein transferred onto PVDF membranes (Millipore, Massachusetts, USA). The membranes were blocked with 5% skimmed milk at room temperature, and then incubated with primary antibodies overnight at 4°C. Antibodies against Cyclin D1 (1:1000, Abcam), CDK4 (1:1000, Abcam), Fibronectin (1:1000, Abcam), Collagen IV (1:400, Abcam), JunD (1:1000, Abcam), Sp1 (1:1000, Millipore), β-actin (1:5000, Abcam). The second day, the membranes were incubated with goat anti-rabbit HRP-IgG secondary antibodies (1: 5000, Beyotime, Shanghai, China) for 90 min. The immune response bands were detected by an enhanced chemiluminescence (ECL) system (Millipore). The semi-quantified data of bands were using ImageJ software.

### Dual-Luciferase Reporter Assay

Based on the experimental design for Sp1 binding sites, the 100bp sequence before and after the P1, P2, or P3 sites of Rmrp promoter was synthesized and constructed into pGL3-basic luciferase reporter vector (WT). Meanwhile, the binding sites of P1, P2, or P3 sequence were mutant and constructed into pGL3-basic vector (Mut). According to the manufacturer’s protocol, the above vectors were co-transfected with pcDNA3.1-Sp1 vector, respectively. To verify the seed-sequence complementation between miR-1a-3p and Rmrp, the wild-type (wt) or seed-sequence mutant (mut) of full-length Rmrp was constructed by Ribo Biotech (Guangzhou, China), and co-transfected with mimics NC or miR-1a-3p or in MC, respectively. Meanwhile, MC and the over-expressed Rmrp of MC were co-transfected with the wt-JunD 3’ UTR reporter plasmid or mut-JunD 3’ UTR and mimics NC or miR-1a-3p (Ribo Biotech, Guangzhou, China). After 48 h transfection, the activities of firefly and Renilla luciferase were measured by the Dual-Glo Luciferase Assay System (Promega, WI, USA).

### Chromatin Immunoprecipitation Assay

The detailed method was performed as previously reported (20). In brief, according to the protocol of Millipore EZ-ChIP kit (Millipore), the protein-DNA complex were combined with anti-Sp1 antibody (Millipore) overnight at 4°CC. 1% the protein-DNA complex was reserved as input control. Normal rabbit IgG (Millipore) was used as the negative control of anti-Sp1 antibody. Immunoprecipitate complexes were collected with protein G Sepharose. After washing and extracting, the purified genomic DNA fragments were analyzed by qRT-PCR. All the primer sequences were displayed in **Supplementary Table 2**.

### RNA Immunoprecipitation Assay

The detailed method was performed as previously reported (21). In brief, EZ-Magna RIP kit (Millipore) was performed to the RIP assays. The Ago2 antibody (Abcam) was used to combine the Ago2-dependent complex and normal rabbit IgG (Millipore) was used as negative control. One tenth of the lysate was reserved as input control. The enrichment levels of miR-1A-3p and Rmrp were analyzed by qRT-PCR.

### Statistical Analysis

All statistical analyses were conducted by GraphPad Prism 7.0 (GraphPad Software, San Diego, CA). The data are presented as mean ± standard deviation (SD). Student’s t-test or one-way ANOVA were used for statistical comparisons as indicated. p< 0.05 was considered as statistically significant.

## Results

### LncRNA Rmrp Is Significantly Up-regulated in DN

**Figure 1:**
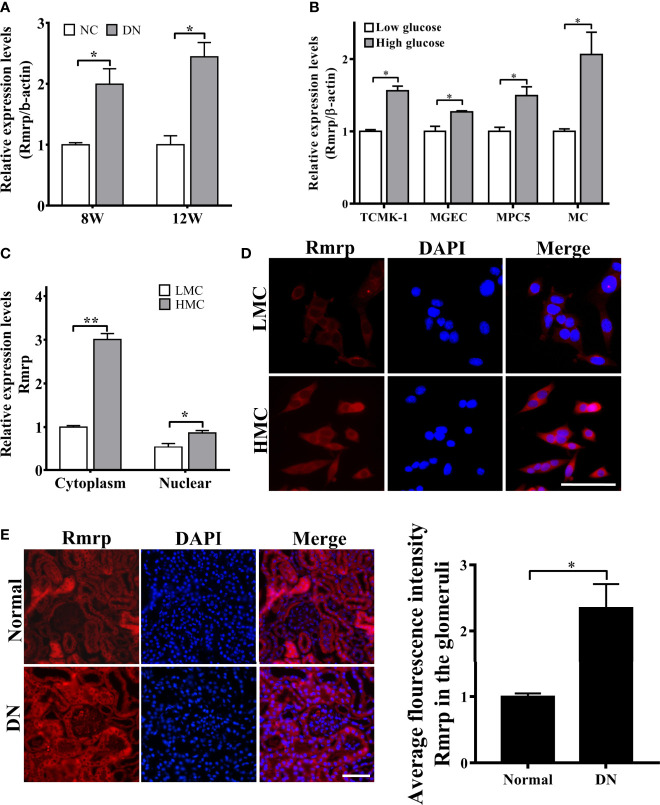
Rmrp is up-regulated in DN. **(A)** Relative expression of Rmrp was identified by quantitative real-time PCR (qRT-PCR) in the renal tissues of db/db DN mice and normal mice at 8 or 12 weeks of age (n=5/group). **(B)** Relative expression of Rmrp was determined by qRT-PCR in four types of renal cells cultured under low (5.5 mmol/L glucose) or high glucose (25 mmol/L glucose) conditions. **(C)** Nuclear-cytoplasmic fractionation assay showed that Rmrp was mainly observed in the cytoplasm of H-MCs by qRT-PCR; β-actin was the cytoplasm control and U6 was the nucleus control. **(D)** FISH was performed to observe the cellular location of Rmrp (red) in LMC and HMC (Scale bar: 50 μm); the nuclei were stained with DAPI. The nuclei were stained with DAPI. **(E)** The localization of Rmrp (red) was observed by FISH and quantitative analysis in the renal tissue of db/db DN mice and the normal group (Scale bar: 50 μm). Data were represented as the mean ± SD of three independent experiments; *p < 0.05, **p < 0.01.

In order to further explore the expressions of Rmrp in DN progression, qRT-PCR was applied to determine the expression level of Rmrp in 8W and 12W DN mice and the age-matched normal mice, which were conserved in our laboratory (16). The results demonstrated that Rmrp was significantly up-regulated in the kidney of DN mice (**Figure 1A**). To observe the effect of glucose on the expression of Rmrp in glomerular endothelial cell (MGEC), glomerular mesangial cells (MC), glomerular podocyte cells (MPC5) and renal tubular epithelial cells (TCMK-1), we cultured four types of cells in low glucose levels (5.5 mM glucose and 19.5 mM mannitol) or high glucose levels (25 mM) medium for 48 hr. In high glucose levels, the expression of Rmrp was the most up-regulated in MC (**Figure 1B**). Subsequently, RT-PCR and FISH assays were used to detect the subcellular localization of Rmrp. The qRT-PCR results showed that Rmrp was markedly higher in the cytoplasm of high glucose-induced MC (HMC) than in the cytoplasm of low glucose induced MC (LMC) (**Figure 1C**). Similarly, FISH assays revealed that Rmrp was mainly localized in the cytoplasm of MC and in the glomerulus of DN mice (**Figures 1D, E**). These data suggested that lncRNA Rmrp might participate in the mesangial cell dysfunction of DN.

### Rmrp Promotes Proliferation and Fibrosis in MC

To explore the biological effects of Rmrp in DN, we constructed the overexpression lentivirus of Rmrp and designed three Rmrp siRNAs (siRmrp-1, siRmrp-2, and siRmrp-3). The results of qRT-PCR showed that the expression of Rmrp in LMC transfected with Rmrp lentivirus was significantly up-regulated (**Supplementary Figure 1A**). Meanwhile, the knockdown effect of siRmrp-1 and siRmrp-2 were better to that of siRmrp-3 in HMC (**Supplementary Figure 2B**). Thus, siRmrp-1 and siRmrp-2 were selected for further study.

**Figure 2:**
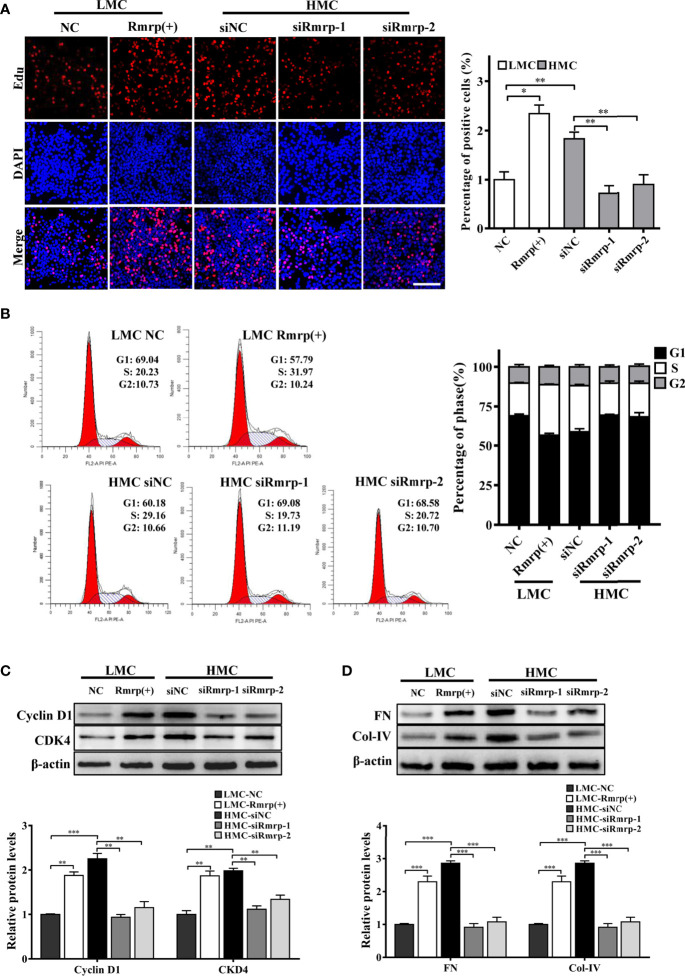
Rmrp regulated cell proliferation and fibrosis in MC. **(A)** cells proliferating were performed by EdU after transfection of Rmrp siRNA in HMC, or over-expression Rmrp in LMC (Scale bar, 100 μm). **(B)** Cell cycle analysis was performed using flow cytometry in MC transfected with si-Rmrp and Rmrp (+). **(C)** The expressions of cell-cycle-related proteins Cyclin D1 and Cyclin-dependent kinase 4 (CDK4) regulated by Rmrp were analyzed by western blot. **(D)** The fibrosis-related protein expression of Collagen IV (Col-IV) and Fibronectin (FN) in LMCs transfected with Rmrp (+) and in HMC transfected with siRmrp was analyzed by western blot. Data were represented as the mean ± SD of three independent experiments; *p < 0.05, **p < 0.01, and ***p < 0.001.

In our previous studies, we found that high glucose possess MC proliferation and fibrosis (21). Thus, we wondered to know the role of Rmrp in these findings. The EdU assays, cell cycle analysis and western blot were used to detect cell growth predominance. Through EdU assay and quantitative analysis, the proportion of cell proliferation was increased after overexpression of Rmrp in LMC, and was attenuated in HMC which knock down of Rmrp (**Figure 2A**). The flow cytometry analysis displayed that the percentage of G1 phase was declined and S phase ratio was raised after overexpression of Rmrp in LMC, while the percentage of cells were arrested in G1 phase and less cells were progressed through to the S phase after silencing Rmrp in HMC (**Figure 2B**). Furthermore, western blot assay demonstrated that overexpression of Rmrp increased the expression of G1 phase-related proteins Cyclin-dependent kinase 4 (CDK4) and Cyclin D1 in LMC, while the protein levels of CDK4 and Cyclin D1 was reversed once Rmrp was restrained in HMC (**Figure 2C**). Similarly, we found that Collagen IV (Col-IV) and Fibronectin (FN) as the renal fibrosis biomarkers, were significantly enhanced after overexpression of Rmrp in LMC and were declined in HMC transfected with Rmrp siRNA (**Figure 2D**). Collectively, our results showed that high Rmrp expression drove cell proliferation and fibrosis in MCs, whereas inhibition of Rmrp alleviated cell proliferation and fibrosis.

### Transcription Factor Sp1 Activated Expression of lncRNA Rmrp in MC

**Figure 3:**
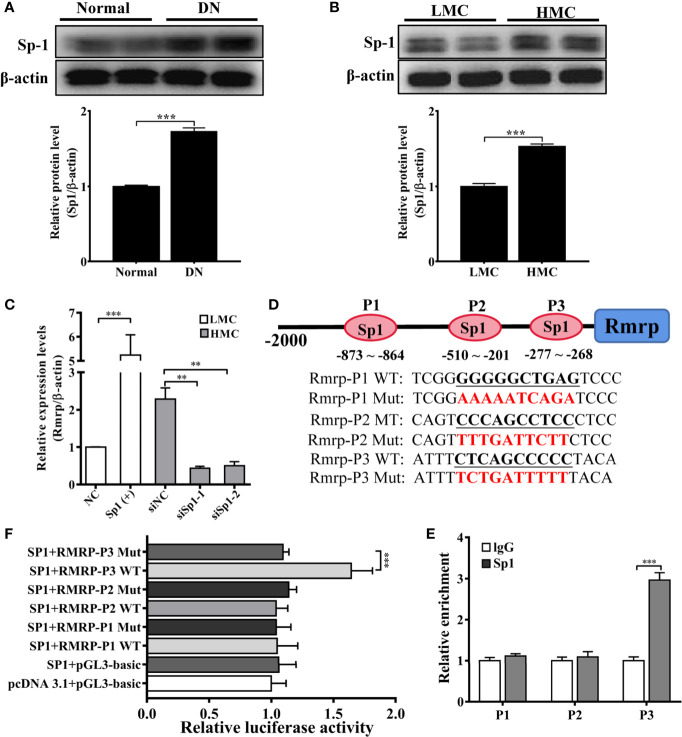
Sp1 enhance the Rmrp expression in the transcription. **(A)** Western blot and quantitative analysis of Sp1 in the kidneys of DN mice and normal mice. **(B)** Western blot and quantitative analysis of Sp1 levels in HMC and LMC. **(C)** The expression of Rmrp was detected by qRT-PCR in HMC with transfection of Sp1 siRNA or in LMC with Sp1 overexpression plasmids. **(D)** Schematic illustration revealed the three predicted positions of SP1 and the wild type (WT) and mutant (Mut) sequences in -2000bp Rmrp promoter. **(E)** The relative luciferase activities were detected in cells co-transfected with Sp1 overexpression plasmids and luciferase reporter plasmids containing wild type or mutant Rmrp promoter sequence. **(F)** ChIP-qPCR assays were performed to determine the potential Sp1 binding site to the promoter of Rmrp in MC; IgG was used as a negative control. Data were represented as the mean ± SD of three independent experiments; *p < 0.05, **p < 0.01, and ***p < 0.001.

As a preceding study of the whole transcriptome analysis in DN mice, we find some transcription factors are abnormal expression in the progression of DN (16). The study indicates that transcription factors play an important role in lncRNA dysfunction (22). To explore the potential transcriptional regulators of Rmrp promoter in MC, the binding sites of potential transcription factors were scanned by online bioinformatics tool (http://jaspar.genereg.net). After analyzing the potential transcription factors of Rmrp promoter and the aberrant transcription factors in our previous RNA-seq data of DN mice (16), the Specificity protein 1 (Sp1) was found 8 putative binding sites on Rmrp promoter. To explore the effects of Sp1, the expression of Sp1 were evaluated in the kidney of DN mice and HMC by western blot. The results showed that Sp1 protein was significantly up-regulated in the kidney of DN mice and HMC (**Figures 3A, B**). Therefore, we hypothesized that elevated Sp1 might regulated the expression level of Rmrp in the transcription, and selected 3 binding sites with scores > 9 for further study. Sp1 expressing plasmids or siRNA were constructed respectively, and qRT-PCR showed that the expression level of Sp1 was changed accordingly (**Supplementary Figure 2**). Correspondingly, the results revealed that Sp1 overexpression vectors significantly increased the expression level of Rmrp in MC (**Figure 3C**), whereas the Sp1 siRNA annihilated the level of Rmrp (**Figure 3D**). Furthermore, we want to explore whether the Sp1 could bind on the potential sites to accelerate the transcription activity of Rmrp, thus the 3 binding sites of the wild type (WT) or mutant (Mut) Rmrp sequence were integrated into luciferase reporter plasmids, respectively (**Figure 3B**). The results revealed that the luciferase activity of the P3 sites but not mutant P3 sites was promoted by Sp1 in Rmrp promoter (**Figure 3E**). Similarly, the results of ChIP-qPCR further confirmed that Sp1 could bind to the P3 sites in the promoter of Rmrp (**Figure 3F**). These data demonstrated that transcription activity of Rmrp was mediated by transcription factor Sp1.

### LncRNA Rmrp Acts as a Sponge for miR-1a-3p

**Figure 4:**
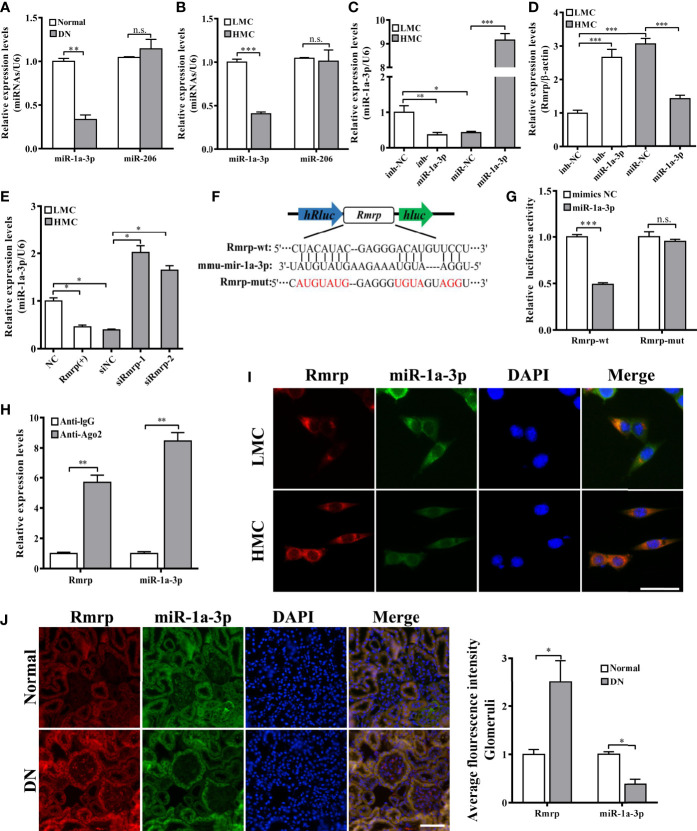
Rmrp functioned as a ceRNA by sponging miR-1a-3p. **(A)** The expression of miR-1a-3p and miR-206 were detected by qRT-PCR in the renal tissues of db/db DN mice and normal mice (n = 5/group). **(B)** The expression of miR-1a-3p and miR-206 were detected by qRT-PCR in LMC and HMC. **(C)** The expression levels of miR-1a-3p were detected by qRT-PCR after transfection with miR-1a-3p mimics and inhibitor in MCs. **(D)** The expression of Rmrp regulated by miR-1a-3p mimics and inhibitor were detected by qRT-PCR. **(E)** The expression of miR-1a-3p were detected in LMCs transfected with Rmrp (+) and in HMC transfected with siRmrp. **(F)** Schematic illustration revealed the base complementation of miR-1a-3p with Rmrp and mutant (Mut) sequences. **(G)** Luciferase assay was used to test relative luciferase activities of Rmrp in MC co-transfected with the indicated miR-1a-3p or control vector. **(H)** Anti-AGO2 RIP was performed in HMC and the RNA levels of Rmrp and miR-1a-3p were determined by qRT-PCR. **(I)** The co-localization of Rmrp and miR-1a-3p was observed in LMC and HMC (Scale bar, 50 μm) by FISH assay. **(J)** The co-localization of Rmrp and miR-1a-3p was observed in the renal tissues of db/db DN mice and normal mice by FISH assay and quantitative analysis (Scale bar, 50 μm). Data were represented as the mean ± SD of three independent experiments; *p < 0.05, **p < 0.01, and ***p < 0.001. ns, no significant.

LncRNAs can function as miRNA sponges to regulate target gene expressions. In order to explore the potential molecular mechanism of Rmrp, the miRcode database (http://mircode.org/index.php), miRanda database (http://www.microrna.org) and RNAhybrid (http://bibiserv.techfak.uni-bielefeld.de/rnahybrid) were applied for bioinformatics analysis, and the possibility of Rmrp and miRNA was determined binding site. The data suggested that miR-1a-3p and miR-206 are potential downstream targets of Rmrp. Based on the prediction, the expression of miR-1a-3p and miR-206 were evaluated in the renal tissue and MC by qRT-PCR. Strikingly, results revealed that the expression level of miR-1a-3p was decreased in the kidney of DN mice and HMC (**Figures 4A, B**). Therefore, we supposed that miR-1a-3p could be sponged by Rmrp. Next, miR-1a-3p mimics or inhibitor changed the down-regulation or up-regulation of miR-1a-3p in MC, respectively (**Figure 4C**). Correspondingly, the expression levels of Rmrp was improved or restrained with miR-1a-3p mimics or inhibitor (**Figure 4D**). In addition, miR-1a-3p was silenced after overexpression of Rmrp in LMC, and was enhanced in HMC which knock down of Rmrp (**Figure 4E**). Therefore, the expression of Rmrp and miR-1a-3p should act as a bidirectional repression.

To explore the exact binding site, the dual-luciferase reporter assays were applied to evaluate the interaction between Rmrp and miR-1a-3p. The WT or Mut Rmrp sequence which was complementary to miR-1-3p was integrated into dual luciferase reporter plasmids, respectively (**Figure 4F**). The data showed that miR-1a-3p mimics significantly suppressed the luciferase intensity of WT Rmrp sequence, but did not reduce the luciferase intensity of Mut Rmrp (**Figure 4G**). This indicates that Rmrp may directly bind to miR-1a-3p. Moreover, miRNAs could bind their target gene to regulate RNA expression through RNA-induced silencing complex (RISC) in an Ago2-dependent manner (21). To further identify the interactive binding of Rmrp with miR-1a-3p, the antibody against Ago2 was used to conduct RIP assay, and IgG as a negative control. Results revealed that both Rmrp and miR-1a-3p were abundantly pulled down by Ago2 antibody rather than IgG control (**Figure 4H**). Furthermore, FISH assay proved that miR-1a-3p was co-localized with Rmrp in the glomerulus of DN mice and in the cytoplasm of MC (**Figures 4I, J**). These data confirmed that Rmrp interacted with miR-1a-3p by directly targeting way in an Ago2-dependent manner. To summarize, Rmrp might play a sponge of miR-1a-3p in MC.

### MiR-1a-3p Reverses the Effects of lncRNA Rmrp in MC

**Figure 5:**
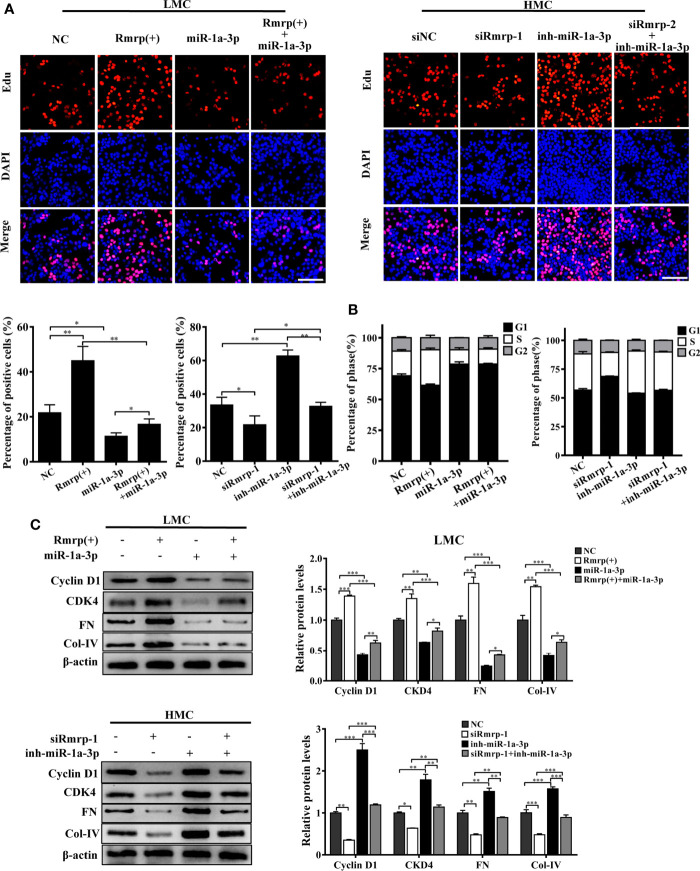
miR-1a-3p reversed the proliferation and fibrosis of MC induced by Rmrp. **(A, B)** The cells proliferating and cell cycle analysis were performed to detect the competition effects of Rpph1 and miR-1a-3p by EdU(Scale bar, 100 μm) and flow cytometry, respectively. **(C)** The expressions level of Cyclin D1, CKD4, Col-IV and FN was analyzed after regulation the level of Rmrp, miR-1a-3p or both of them by western blot. Data were represented as the mean ± SD of three independent experiments; *p < 0.05, **p < 0.01, and ***p < 0.001.

In order to prove the interaction between Rmrp and miR-1a-3p in MC, Rescue experiments were performed by co-transfecting miR-1a-3p mimic or miR-1a-3p inhibitor with Rmrp vector or si-Rmrp. The EdU assay showed that the up-regulated Rmrp increased the proliferation rate of LMC, while these effects were blocked by miR-1a-3p mimics (**Figure 5A**). Meanwhile, reduced HMC proliferation by knockdown of Rmrp was regained with miR-1a-3p inhibitors. The flow cytometry analysis displayed that the percentage of S phase was raised after overexpression of Rmrp in LMC, while these phenomenon were repressed by miR-1a-3p mimics. Although t the percentage of S phase were down-regulate by silence of Rmrp, these changes were blocked by miR-1a-3p inhibitor (**Figure 5B**). Additionally, western blot results showed that the miR-1a-3p mimics significantly reduced the expression Cyclin D1, CKD4, FN and Col-IV promotion caused by the up-regulation of Rmrp in LMC, while miR-1a-3p inhibitors could offset the inhibiting effect of siRmrp in the expression Cyclin D1, CKD4, FN and Col-IV of HMC (**Figure 5C**). Therefore, these experiments demonstrated that Rmrp regulated the proliferation and fibrosis of MC by acting as a ceRNA to sponge miR-1a-3p.

### JunD Is a Direct Target of miR-1a-3p and Promotes Proliferation and Fibrosis Through Rmrp/miR-1a-3p/JunD Axis in DN

**Figure 6:**
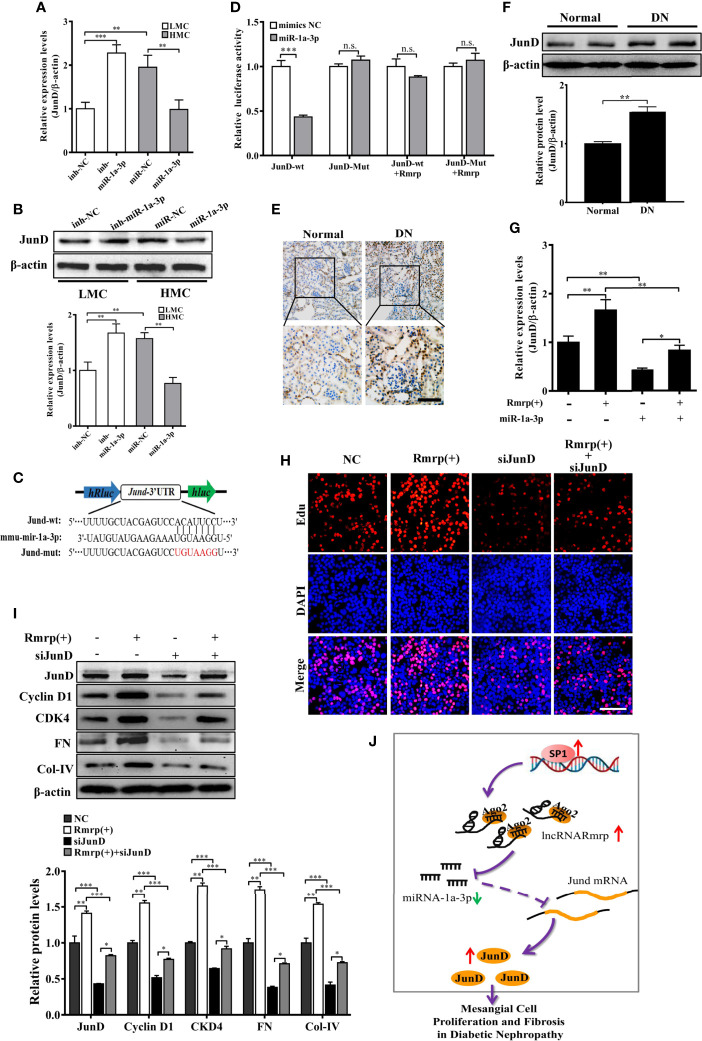
Rmrp regulated the proliferation and fibrosis of MC *via* miR-1a-3p/JunD signaling. **(A, B)** The relative expression of JunD was detected after transfection with miR-1a-3p mimics and inhibitor by qRT-PCR and western blot. **(C)** The schematic model revealed the JunD 3’ -UTR wide type (WT) and mutant (Mut) luciferase reporter vectors. **(D)** The luciferase reporter assays showed that miR-1a-3p directly inhibited the luciferase activity of JunD 3’ -UTR WT, but lost efficacy when the Rmrp was up-regulated. **(E, F)** IHC analysis and western blot assessed JunD expression in the renal tissues of DN and normal mice (Scale bar, 50 μm). **(G)** The relative expression of JunD in MC after transfection with Rmrp expression vector or/and miR-1a-3p mimics by qRT-PCR. **(H)** The cells proliferating were performed to detect the competition effects of Rmrp and JunD siRNA by EdU (Scale bar, 50 μm). **(I)** The protein expression of JunD, Cyclin D1, CKD4, Col-IV and FN was analyzed after regulation the level of Rmrp expression vector, JunD siRNA or both of them by western blot. **(J)** The schematic diagram illustrates the mechanism of Rmrp mediated by Sp1 to mesangial cell proliferation and fibrosis through Rmrp/miR-1a-3p/JunD axis in DN. Data were represented as the mean ± SD of three independent experiments; *p < 0.05, **p < 0.01, and ***p < 0.001. ns, no significant.

According to the TargetScan (http://www.targetscan.org/vert_72/), TarBase V.8 (http://carolina.imis.athena-innovation.gr/diana_tools/web/index.php?r=tarbasev8%2Findex), and our previous RNA-seq data of DN mice (16), Jun D proto-oncogene (JunD) contains conserved target sites of miR-1a-3p. As known, JunD plays a crucial regulator of cell proliferation (23) and fibrogenesis (23) in the progression of disease. Therefore, the qRT-PCR and western blot analysis exhibited that miR-1a-3p inhibitor could enhanced the RNA and protein level of JunD in LMC, while miR-1a-3p mimics significantly reduce the RNA and protein level of JunD in HMC (**Figures 6A, B**). Next, we constructed dual-luciferase reporter plasmids embedding the wild and mutant JunD 3’ -untranslated regions (UTRs), respectively (**Figure 6C**). Using dual-luciferase assay, miR-1a-3p mimics strongly reduced the fluorescent intensity of JunD 3’-UTR WT luciferase reporter but not that of mutants (**Figure 6D**). Interestingly, we found that the fluorescent activity of JunD 3’-UTR WT reporter plasmids was not influenced by miR-1a-3p mimics in MC when Rmrp was up-regulated. These data suggest that miR-1a-3p directly regulated the expression of JunD.

To clarify whether the effect of Rmrp on proliferation and fibrosis was the interaction with JunD, the JunD expression was detected by evaluated with immunohistochemistry (IHC) and western blot in DN mice. The results revealed JunD was increased in the kidney of DN mice compared with matched the normal tissues (**Figures 6E, F**). Meanwhile, the qRT-PCR data showed that the enhancive effect of Rmrp overexpression on JunD mRNA level was rescued by miR-1a-3p mimics. The results showed that Rmrp and miR-1a-3p could be working together to regulate the expression of JunD (**Figures 6G**). Additionally, we designed three JunD siRNAs (siJunD-1, siJunD-2, and siJunD-3). The qRT-PCR and results showed the knockdown effect of siJunD-1 was the best in HMC (**Supplementary Figure 3**). Thus, siJunD-1 was selected for further study. Next, the EdU assay revealed that overexpression of Rmrp was beneficial to MC proliferation, while these effects were suppressed by JunD siRNA (**Figure 6H**). Similarly, the results of Western blot showed that overexpression of Rmrp increased the expression Cyclin D1, CKD4, FN and Col-IV, whereas knockdown of JunD could abolish the effects, respectively (**Figure 6I**). These results indicated that Rmrp can act as a sponge for miR-1a-3p by regulating JunD, thereby promoting proliferation and fibrosis in MC. As shown in **Figure 6J**, we found that Rmrp was significantly upregulated by Sp1 and Rmrp functioned as amiR-1a-3p sponge to positively regulate JunD expression, thereby leading to mesangial cell proliferation and fibrosis in DN.

## Discussion

It is well known that MC organizes the extracellular matrix production and degradation to regulate glomerular structure and function in DN (24). However, the complex mechanisms of MC activation and the underlying effects have yet to be explored in DN. In recent years, more and more evidence has demonstrated that the abnormal lncRNAs could participate in the expression and/or function of genes, and modulate the progression of DN. However, the potential roles of lncRNAs in the progression of DN are still unknown.

It was previously shown RMRP was important for mitochondrial DNA replication as the RNA primer and essential for enzymatic activity (25), but how Rmrp work in the cross regulation between nucleus and mitochondria remain largely unclear. Interestingly, in the generation of human disease, Rmrp was initially identified to be essential for cartilage-hair hypoplasia (26). Since RMRP was translocated to the cytoplasm for mitochondrial RNA processing activity (27), it was reported that the elevation in the expression of RMRP could promote the progression of diseases, such as lung Cancer (28), atherosclerosis (29) and coronary heart disease (30). Moreover, the functions of RMRP activated the abundant signaling pathways, which included PI3K/AKT/mTOR (30), NF-κB (31), TACR1/Erk1/2 (32) and other. It was suggested RMRP should play an important role/part in the distinct mechanisms of tumor and other diseases. By using high-throughput RNA-seq, we had found the up-regulated expression of lncRNAs Rmrp in the kidney of DN mice (16, 17). Nevertheless, the function of abnormal RMRP in the progression of DN is poorly known. In this report, we demonstrated that the lncRNA RMRP was significantly upregulated in the renal tissues of DN mice and in high glucose-induced MC damage *in vitro*. Rmrp promoted the proliferation of MC as well as the expression of CKD4 and Cyclin D1, and also fortified the overproduction of Collagen IV and Fibronectin, which were both critical for ECM components. Inhibition of Rmrp significantly reversed these above impacts, implying that the Rmrp could contribute to the progression of DN.

To further explore the regulatory mechanism of the abnormal Rmrp expression in DN, we focused on transcription factors which were contributed to lncRNAs dysregulation. By bioinformatics analyses, Sp1 was found to possess potential binding sites in the Rmrp promoter. Sp1 is one such ubiquitous and multifunctional transcription factor from the Sp/Kruppel-like family (KLF) (33), and interact with GC-rich motifs of promoter regions to execute transcriptional activation function. Sp1 was involved in the dysregulation of multiple lncRNAs. For instance, Chen et al. reported that lncRNA ZFAS1 expression was increased in colorectal cancer and was activated by Sp1 (34). Our result supported that the expression level of Sp1 had been activated by glucose and in the DN. Subsequently, the overexpression of Sp1 increased the level of Rmrp and Sp1 siRNA inhibited the expression of Rmrp. Furthermore, we used bioinformatics assay, Luciferase reporter assays and ChIP assay to confirm that Sp1 could bind to the Rmrp promoter region. These data clearly verified that Rmrp was transcriptionally activated by Sp1 in the progression of DN.

Accumulating data indicates that lncRNAs are emerging as important regulators in gene expression networks by regulate mRNA stability *via* associated miRNAs (35). LncRNAs perform ceRNAs function as miRNA sponges to derepressing miRNA target gene binding (36). For example, it was reported that LINC01619 was downregulated in human DN renal biopsy tissues, and exerted its biological function by binding to miR-27a to act the FOXO1-mediated ER stress and podocyte injury in DN (37). In addition, RMRP might promote cell proliferation, migration, and invasion in non-small-cell lung cancer *via* sponging miR-1a-3p (38). To explore the mechanism of Rmrp in DN, the target miRNAs were analyzed by online databases and qRT-PCR assay. The results revealed that miR-1a-3p was the opposite expression compared with Rmrp expression in DN and there was a bidirectional repression between miR-1a-3p and Rmrp. FISH assay indicated co-localization of Rmrp and miR-1a-3p in the sub cellular level of MC. Furthermore, the direct relationship between Rmrp and miR-1a-3p was further identified by Dual-luciferase reporter assay and RIP assay. It is well known that a single lncRNA can bind on multiple miRNAs to regulate gene expression. Whether Rmrp sponges other miRNAs involved in DN still needs to be explored. Here, in the present study, the results showed that Rmrp should function interact with miR-1a-3p by both directly targeting and Ago2-dependent ways in DN.

Based on the ceRNA hypothesis, miRNAs exert their function by blocking the translation of target genes or inducing mRNA degradation (39). MiR-1a-3pa-5p have been reported be decreased levels as a tumor suppressor and suppresses tumor progression in colorectal cancer (40), glioblastoma (41) and lung cancer (42). In diabetic complications, only a few studies have been reported miR-1a-3p is downregulated in retinal and renal tissues of STZ-induced diabetic mice (43). However, detailed mechanisms in the regulatory process of miR-1a-3p need further elucidation. In this study, we foundmiR-1a-3p was also downregulated in DN mice and glucose-induced MC, and loss of miR-1a-3p in mouse MC improved cell proliferation and fibrosis. Moreover, bioinformatics analysis showed that JunD is a latent target of miR-1a-3p. Subsequently, we confirmed that miR-1a-3p might directly bind to the 3’ -UTR JunD by dual-luciferase assay. Besides, JunD could be modulated by the miR-1a-3p mimics and inhibitor at the mRNA and protein levels. Other studies reported JunD expression was increased in hearts from diet-induced obese mice, and regulated PPARc signaling leading to cardiac damage (44). miR-548d-3p directly targets JunD and loss of miR-548d-3p enhances JunD/RSK3 signaling in the chemotherapy resistant of breast cancer (45). JunD as a member of the transcription factors Activated Protein-1 (AP-1) family, is essential as a major gatekeeper in cell proliferation and fibrogenesis. Studies showed that JunD as a transcription factor could bind to the TRE site of Cyclin D1 promoter to influence Cyclin D1 transcription (46). Meanwhile, JunD could up-regulate the expression of CDK4 through inducing c-MYC (23). In other studies, the data demonstrated that JunD is implicated in the transcriptional regulation of fibrosis (47) and JunD is a mediator of the profibrotic effects of TGF-β (48). The detailed mechanisms in the regulatory process need further elucidation. Herein, our results showed JunD was up-regulated in DN mice and glucose-induced MC. At the same time, we revealed that the upregulation of JunD mRNA and protein could be regulated by overexpression of Rmrp. Subsequently, Rmrp-induced proliferation and fibrogenesis were reversed by co-transfection with JunD siRNA. In sum, our results further suggested that Rmrp serves as a ceRNA formiR-1a-3p to enhance JunD expression and activate proliferation and fibrogenesis in DN through miR-1a-3p/JunD axis.

In summary, Rmrp expression was found to be increased in DN and high glucose induced-MC, and the abnormal re-activation of Rmrp was mediated by transcription factor SP1.Furthermore, we found that Rmrp might sponge miR-1a-3p to release JunD expression and activate cell proliferation and fibrosis during DN. Finally, we hoped that these data would provide a new glimpse of the underlying mechanism and a new prospective therapeutic target for DN.

## Data Availability Statement

The original contributions presented in the study are included in the article/**Supplementary Material**. Further inquiries can be directed to the corresponding author.

## Ethics Statement

The animal study was reviewed and approved by the Ethics Committee of Chongqing Medical University.

## Author Contributions

YS designed the research and supervised the project. HY and JW executed all experiments. HSY and ZZ performed statistical analysis of data. RP was responsible for bioinformatics analysis. DL and HL provided support experimental technical support. YS and ZZ wrote the manuscript. All authors contributed to the article and approved the submitted version.

## Funding

This study was supported by the National Natural Science Foundation of China (No. 81700639, 81770811 and 81970702); the Top-notch Talents Program for graduate students of Chongqing Medical University (BJRC201916 and BJRC201919).

## Conflict of Interest

The authors declare that the research was conducted in the absence of any commercial or financial relationships that could be construed as a potential conflict of interest.

## Publisher’s Note

All claims expressed in this article are solely those of the authors and do not necessarily represent those of their affiliated organizations, or those of the publisher, the editors and the reviewers. Any product that may be evaluated in this article, or claim that may be made by its manufacturer, is not guaranteed or endorsed by the publisher.
