# Supplementary material for: Sp1-Induced lncRNA Rmrp Promotes Mesangial Cell Proliferation and Fibrosis in Diabetic Nephropathy by Modulating the miR-1a-3p/JunD Pathway
Source: Front Endocrinol (Lausanne). 2021 Aug 27;12:690784. doi: 10.3389/fendo.2021.690784 (PMC8429906; doi:10.3389/fendo.2021.690784)
Supplement: Supplementary file 1 [file Image_1.pdf]

Supplementary figure 1

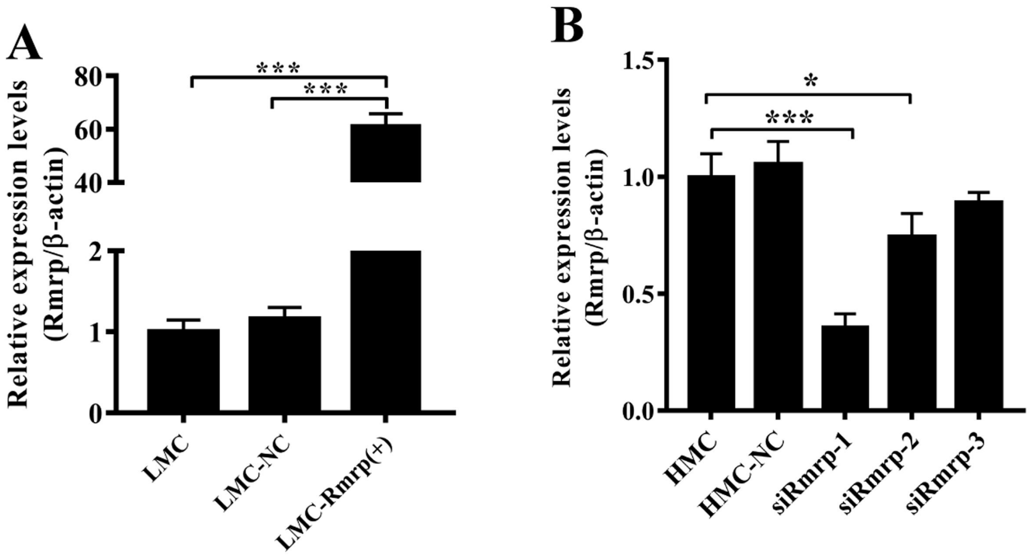

Supplementary figure 1. The expression levels of Rrmp were detected by qRT-PCR after transfection with Rrmp over-expression in L-MC **(A)** or Rrmp small interfering RNA (siRNA) in H-MC **(B)**. Data were represented as the mean ± SD of three independent experiments; *$p < 0.05$ and ***$p < 0.001$.

Supplementary figure 2

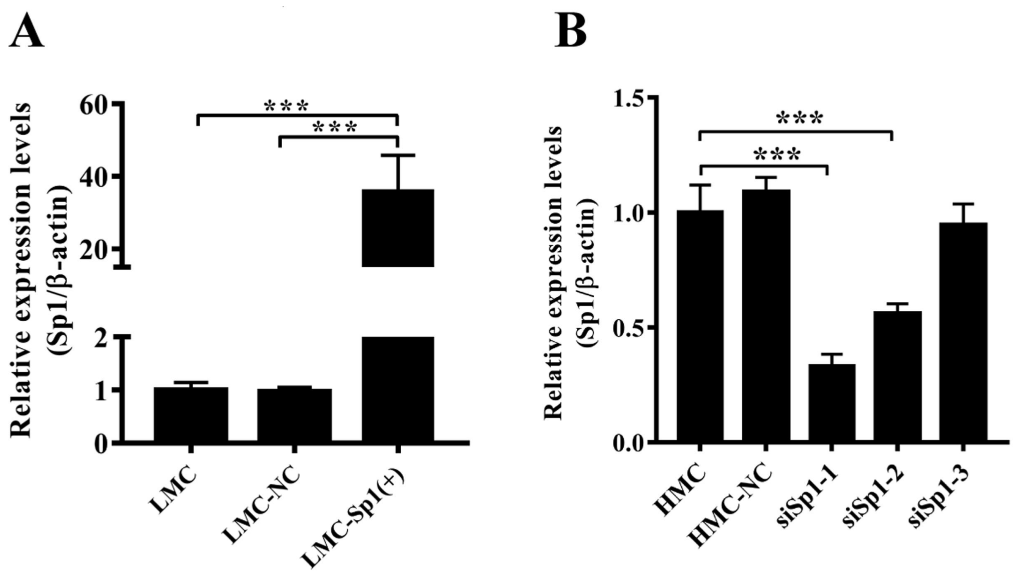

Supplementary figure 2. The expression levels of Sp1 were detected by qRT-PCR after transfection with Sp1 over-expression in L-MC **(A)** or Sp1 siRNA in H-MC **(B)**. Data were represented as the mean ± SD of three independent experiments; ***$p < 0.001$.

Supplementary figure 3

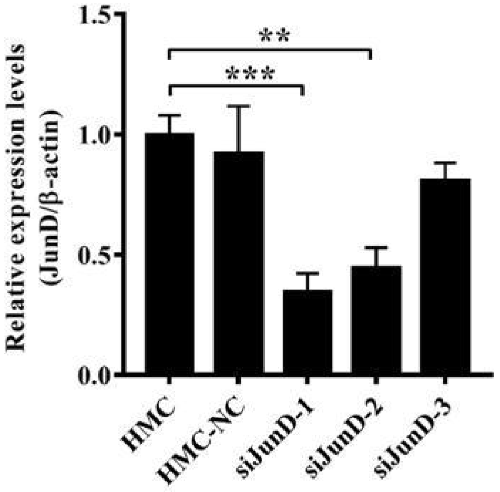

Supplementary figure 3. The expression levels of JunD were detected by qRT-PCR after transfection with Sp1 siRNA in H-MC. Data were represented as the mean ± SD of three independent experiments; **$p < 0.05$ and***$p < 0.001$.
