# Supplementary material for: Sp1-Induced lncRNA Rmrp Promotes Mesangial Cell Proliferation and Fibrosis in Diabetic Nephropathy by Modulating the miR-1a-3p/JunD Pathway
Source: Front Endocrinol (Lausanne). 2021 Aug 27;12:690784. doi: 10.3389/fendo.2021.690784 (PMC8429906; doi:10.3389/fendo.2021.690784)
Supplement: Supplementary file 2 [file Table_1.docx]

| **Supplementary Table 1. sequences of siRNAs** | | |
| --- | --- | --- |
| **Gene** |  | **sequence** |
| miR-1a-3p mimics | Sense | UGGAAUGUAAAGAAGUAUGUAU |
|  | Antisense | AUAUACUUCUUUACAUUCCAUU |
| miR-1a-3p inhibitor | Sense | AUAUAUACUUCUUUACAUUCCA |
| siRmrp-1 | Sense | ACTGTTAGCCCGCCAAGAA |
| siRmrp-2 | Sense | CAGCTCACATAGTGACGCA |
| siRmrp-3 | Sense | GGACATGTTCCTTATCCTT |
| siJunD-1 | Sense | GUUCGCCGAAGGCUUCGUCAATT |
|  | Antisense | UUGACCAAGCCUUCGGCGAACTT |
| siJunD-2 | Sense | CCACGUCAACAGCGGCUGCCATT |
|  | Antisense | UGGCAGCCGCUCUUGACGUGGTT |
| siJunD-3 | Sense | CGCCGGAUCUUGGGCUGCUCATT |
|  | Antisense | UGAGCAGCCCAAGAUCCGGCGTT |
| siSp1-1 | Sense | CCAAUGCCAAUAGUUAUUCAATT |
|  | Antisense | UUGAAUAACUAUUGGCAUUGGTT |
| siSp1-2 | Sense | ACCAACAGAUCAUCCCAAAUATT |
|  | Antisense | UAUUUGGGAUGAUCUGUUGGUTT |
| siSp1-3 | Sense | UCAUGUGUAAUUGGUCAUAUUTT |
|  | Antisense | AAUAUGACCAAUUACACAUGATT |
| Negative control | Sense | UUCUCCGAACGUGUCACGUTT |
|  | Antisense | ACGUGACACGUUCGGAGAATT |

| **Supplementary Table 2. sequences of primers** | | |
| --- | --- | --- |
| **Gene** | **primer** | **sequence** |
| Rmrp | Forward | GCTCGCTCTGAAGGCCTGTT |
|  | Reverse | GTAGCCGCGCTGAGAATGAG |
| JunD | Forward | GAAACGCCCTTCTATGGCGA |
|  | Reverse | CAGCGCGTCTTTCTTCAGC |
| miR-1a-3p | RT | GTCGTATCCAGTGCAGGGTCCGAGGTATTCGCACTGGATACGACATACAT |
|  | Forward | GCGCTGGAATGTAAAGAAGT |
|  | Reverse | GTGCAGGGTCCGAGGT |
| Rmrp-P1 | Forward | GTAACGGTTTCTACAATTTCTC |
|  | Reverse | TGTTCAAGTGTTTGCAGGT |
| Rmrp-P2 | Forward | AGCCCCGTTCTGTGGATTA |
|  | Reverse | TGTCTGCGCCTTTTGGAGT |
| Rmrp-P3 | Forward | GGAGACCACGCCTACATC |
|  | Reverse | GTAGGAACGCTAGGTGGG |
| Sp1 | Forward | AAGGATGCGGCAAAGTAT |
|  | Reverse | CGTCCGAACGTGTAAAGC |
| beta-actin | Forward | ATATCGCTGCGCTGGTCGTC |
|  | Reverse | AGGATGGCGTGAGGGAGAGC |
| U6 | Forward | CTCGCTTCGGCAGCACA |
|  | Reverse | AACGCTTCACGAATTTGCGT |
